# Association of plasma levels of protein-bound advanced glycation end-products and their soluble receptors with bone mineral status in young girls with the restrictive type of anorexia nervosa

**DOI:** 10.1007/s11657-025-01554-z

**Published:** 2025-08-27

**Authors:** Katarína Šebeková, Alexandra Gaál Kovalčíková, Alžbeta Čagalová, Ľubica Tichá, Ľudmila Podracká

**Affiliations:** 1 Institute of Molecular Biomedicine, Faculty of Medicine, Comenius University, Sasinkova 4, 811 08 Bratislava, Slovakia; 2 Department of Pediatrics, Faculty of Medicine, National Institute of Children’s Diseases, Comenius University, Limbova 1, 833 40 Bratislava, Slovakia

**Keywords:** Anorexia nervosa, Bone mineral density, Trabecular bone score, CML, MG-H1, EsRAGE

## Abstract

***Summary*:**

In diabetes- and age-related osteopenia/osteoporosis, a pathogenetic role of advanced glycation end-products and their soluble receptors (RAGE) is implicated. We studied how these compounds relate to bone health in girls with anorexia nervosa. We found that higher levels of endogenous secretory RAGE were associated with poorer bone quality, warranting further research.

**Purpose:**

We explored the association of plasma protein–bound advanced glycation end-products (AGEs) and their soluble receptors with bone mineralization and turnover markers in girls with a restrictive type of anorexia nervosa.

**Methods:**

A total of 102 girls with anorexia nervosa aged 14.0 ± 2.7 years and 29 age-matched healthy controls were included. Plasma levels of chemically defined AGEs (N^ε^-(carboxymethyl)-lysine, methylglyoxal-derived hydroimidazolone-1, and their soluble receptors were determined using the ELISA methods; parathormone, osteocalcin, amino-terminal propeptide of human procollagen type I, carboxy-terminal telopeptide of type I collagen (CTX), and estradiol using the electrochemiluminescence. Girls with AN underwent dual-energy X-ray absorptiometry to assess bone mineral density (BMD) and trabecular bone score (TBS). Multivariate regression was performed using the orthogonal projection to latent structures model.

**Results:**

Girls with anorexia nervosa displayed higher plasma levels of N^ε^-(carboxymethyl)-lysine (by 52%) and methylglyoxal-derived hydroimidazolone-1 (by 34%) than controls, while soluble RAGE levels were similar in both groups. In girls with anorexia nervosa, low levels of nutritional markers, high endogenous secretory RAGE, and high CTX predicted low hip and femoral neck BMD. Low levels of nutritional markers and high bone turnover markers predicted TBS.

**Conclusions:**

To clarify the role of the AGEs/RAGE axis in anorexia nervosa-associated low bone mass, longitudinal studies assessing the dynamic changes of these markers during re-alimentation-induced weight restoration and bone health recovery are needed. In clinical practice, monitoring of the AGEs/sRAGE axis could offer a novel approach for assessing disease status and guiding personalized interventions to mitigate long-term health consequences in patients with AN.

**Supplementary Information:**

The online version contains supplementary material available at 10.1007/s11657-025-01554-z.

## Introduction

Advanced glycation end-products (AGEs) are formed in organisms and heated foods via the classical Maillard reaction (non-enzymatic reaction between reducing sugars and free amino groups of proteins) or through amino group modification by reactive α-dicarbonyls, lipid peroxidation, or glycoxidation [1, 2]. AGE modification alters protein structure and function. The interaction of AGE-modified proteins with cell-surface receptors for AGEs (RAGE) triggers inflammation and production of reactive oxygen species (ROS) [3]. Soluble RAGEs (sRAGE, either proteolytically cleaved from the cell surface (cRAGE) or produced by alternative splicing (endogenous secretory RAGE, esRAGE)) act as decoys, inhibiting AGE/RAGE interaction [4, 5]. The AGE/RAGE axis plays a role in several chronic non-communicable and age-related diseases [6], including osteopenia/osteoporosis [7, 8]. It has been documented that enzymatic crosslinking of bone collagen I at specific sites beneficially affects bone mineralization and strength; on the contrary, non-enzymatic crosslinking via AGEs (e.g., pentosidine) occurs at ubiquitous sites and deteriorates biomechanical bone function [9, 10]. Non-crosslinking AGEs (e.g., N^ε^-(carboxymethyl)-lysine—CML, methylglyoxal (MGO)-derived hydroimidazolone-1—MG-H1) impair bone turnover by affecting osteoblasts and osteoclasts activity through RAGE and induction of oxidative stress [11, 12]. In diabetes and aging, the accumulation of AGEs in bones reduces bone quality [9–12].

Anorexia nervosa (AN) is a severe psychosomatic disease with one of the highest psychiatric mortality rates, primarily affecting adolescent girls and young females [13]. Driven by an obsessive fear of weight gain and distorted body image, the patients correct their perceived flaws by self-starvation and, frequently, excessive exercising [14]. Long-term energy, macro- and micro-nutrient deficiency, and starvation-induced catabolism lead to medical complications affecting all organ systems [15]. Estradiol deficiency due to hypogonadotropic hypogonadism and chronic undernutrition during the crucial bone development period impairs skeletal integrity, making low bone mass an early feature of AN [16, 17].

AN-associated medical complications may worsen due to enhanced oxidative stress resulting from an inadequate intake of essential elements, dietary antioxidants, and enhanced ROS production during starvation [18–20]. In a previous study investigating the same cohort, we confirmed that girls with AN present lower antioxidant and higher oxidatively modified protein levels and elevated AGE-associated fluorescence of plasma (AGE-Fl) than their healthy counterparts [19, 21]. AGE-Fl (a non-specific marker of bulk fluorescent AGEs accumulation [22]) inversely correlated with hip bone mineral density (BMD) *z*-score and bone turnover markers (amino-terminal propeptide of human procollagen type I (PINP), osteocalcin) when amenorrhoeic girls with AN and the controls were evaluated together [21]. In another study, young females with low bone mass also had higher plasma AGE levels than those without osteopenia or osteoporosis [23]. Associations of chemically defined AGEs or sRAGE levels with osteopenia or osteoporosis are described in menopausal women or elderly subjects with or without diabetes and are equivocal (reviewed in [7]). To our knowledge, no data exist on their plasma levels or association with bone mineralization markers and bone turnover in patients with AN.

CML and MG-H1 are the most abundant AGEs in plasma or bone [24, 25]. To confirm that the higher AGE-Fl in girls with AN reflects the accumulation of AGEs, we compared CML and MG-H1 plasma levels in our patients with healthy controls. Additionally, we determined esRAGE and cRAGE levels. We hypothesized that girls with AN display higher plasma AGE and lower sRAGE levels than their healthy peers and that in patients, high AGE and low sRAGE levels relate to worse bone mineral status determined via dual-energy X-ray absorptiometry (DEXA).

## Methods

### Study population

We analyzed data from an observational study on oxidative status in girls with AN [19], including all healthy controls and 102 of the 111 originally enrolled patients with sufficient stored plasma for AGE and sRAGE measurements. Forty-one patients (but not the controls) were also part of a previous study on oxidative markers and bone mineral status in amenorrhoeic girls with AN [21].

A detailed diagnostic procedure is provided in the Supplementary file. Briefly, females aged 10 to 19 years with a first diagnosis of restrictive type of AN were included. All girls were submitted to the ward of the Department of Pediatrics at the National Institute of Children’s Diseases (NICD) in Bratislava between October 2016 and February 2020 for differential diagnostics of severe body weight loss. Anorexia nervosa was diagnosed according to the criteria of the *Diagnostic and Statistical Manual of Mental Disorders* (5 th Edition) [14]. Clinical symptoms of anorexia manifested on average 11.7 ± 11.3 months before hospitalization (range 1.7–60.8 months). Twenty-four (23.5%) patients were premenstrual, sixty-one (59.8%) had secondary amenorrhea (a mean duration: 11.3 ± 8.7 months), seven (6.9%) had primary amenorrhea, and 10 (9.8%) patients had a period. Seventeen girls suffered traumatic bone fractures in childhood, all before they started losing weight, and longer than 6 months before the DEXA scanning.

The control group consisted of 29 healthy girls aged 10–18 years without any history of eating or psychiatric disorders. Controls were recruited via primary care pediatricians practicing within the NICD premises.

For both groups, neurological, gastrointestinal, endocrinologic, acute or chronic inflammatory diseases, diseases potentially affecting bone formation, and hormonal contraception were exclusion criteria.

The study was conducted following the guidelines of the Declaration of Helsinki after the Ethics Committee of the NICD, Bratislava, Slovakia, approved the protocol. Written informed consent was obtained from participants’ legal guardians in addition to the participants’ verbal assent.

### Data collection

In girls with AN, anthropometric measurements, blood withdrawal, and DEXA scans were performed within the first week after admission to the hospital, before the diagnosis of AN, and before initiating any re-alimentation therapy. Control subjects underwent anthropometric measurement and blood withdrawal during regular check-ups at the pediatrician’s office. DEXA scans were not taken.

### Anthropometric measurements

Trained nurses measured height (to the nearest 0.1 cm) and body weight (to the nearest 0.1 kg) using a wall-mounted stadiometer and electronic scales, respectively (Seca, Hamburg, Germany). Body mass index (BMI, kg/m^2^) was calculated. Age- and sex-specific reference data from Slovak children and adolescents were used to express each participant’s standard deviation scores (SDS) [26].

### Bone mineral density and body composition assessment

In patients with anorexia, BMD (g/cm^2^) and bone mineral content (BMC, g) of the lumbar spine (L1–L4), left hip, left femoral neck, lumbar spine trabecular bone score (TBS), and lean and fat mass (kg) were determined using the DEXA scanner (Horizon QDR instrument, Hologic Inc., Danbury, CT, USA). Details of procedures are given in the Supplementary file. Data were evaluated using the pediatric software, comparing the obtained data with those from age- and sex-matched reference populations expressed as a *z*-score. BMD *z*-score ≤ − 2 SD was defined as “below the expected range for age” [21]. TBS *z*-score was classified according to Kalkwarf et al. [27].

### Blood sampling, laboratory analyses, and calculations

The procedures are described in detail in the Supplementary file. Briefly, blood samples were obtained by venipuncture of the cubital vein in the morning hours, after at least 8 h of fasting. Routine blood chemistry analyses (plasma glucose, creatinine, uric acid, albumin, total cholesterol, triacylglycerols, calcium, phosphate concentrations, and alkaline phosphatase activity), determinations of 25-OH vitamin D, parathormone, osteocalcin, PINP, carboxy-terminal telopeptide of type I collagen (CTX), estradiol, and insulin-like growth factor-1 (IGF-1) levels were performed in the central laboratory. Aliquots of plasma samples were stored at − 20 °C pending analyses at the Institute of Molecular Biomedicine, where plasma cystatin C, CML, MG-H1, sRAGE, esRAGE, and AGE-Fl were determined. The concentration of cRAGE was calculated as sRAGE minus esRAGE. Plasma cystatin C was used to estimate the glomerular filtration rate (eGFR) via Schwartz equation [28].

Only glucose, creatinine, uric acid, albumin, cholesterol, triacylglycerols, cystatin C, AGE-Fl, CML, MG-H1, sRAGE, and esRAGE levels were determined in the controls.

### Statistical analysis

The sample size was based on detecting a 20% difference in AGE-Fl at *α* = 0.05 with 80% power and a 0.28 enrollment ratio, yielding groups of 29 and 102 probands. Data distribution was assessed using the Shapiro–Wilk test. Normally distributed data is presented as the mean ± SD; skewed data as the median and interquartile range. Controls and patients were compared using the two-sided Student’s *t*-test for independent samples after checking the equality of variances with Levene’s test, or the Mann–Whitney *U* test, as appropriate. Trends across menstrual status categories (premenstrual, primary amenorrhea, secondary amenorrhea, menstruating) were compared by one-way ANOVA with Bonferroni correction or Kruskal–Wallis with Dunn’s tests. Spearman’s or Pearson’s correlations were calculated. The level of statistical significance was set at *p* < 0.05. Analyses were performed using the SPSS v. 23.0 statistical software (IBM, Armonk, NY, USA).

In patients with AN, multivariate analyses were performed using the SIMCA v. 18.2 software (Sartorius Stedim Data Analytics AB, Umea, Sweden). Principal component analysis (PCA) was used to explore the sample distribution without classification. Hotelling’s T2 and DmodX tests were applied to identify potential outliers. The multivariate regression of independent variables on BMD was performed using the orthogonal projections to latent structures (OPLS) model. Lean mass, fat mass, esRAGE, cRAGE, CML, MG-H1, AGE-Fl, CTX, PINP, IGF-1, alkaline phosphatase, phosphate, 25-OH vitamin D, and estradiol were entered as explanatory (independent) variables. The scores plot, loadings plot, the variance (*R*^2^) described by the model, the predictivity of the model (*Q*^2^), and the variables important for the projection (VIPs) were evaluated. Variables with VIP ≥ 1 were considered significant predictors. The models were validated using the permutation test.

Details on statistical analyses are given in the Supplementary file.

## Results

### Descriptive statistics

Cohort characteristics are given in Table 1. Girls with AN presented with lower body weight, body weight SDS, BMI, and BMI SDS (forty of them (39%) were severely undernourished: BMI SDS ≤ − 2 SDS), and displayed lower creatininemia, albuminemia, and uricemia but higher triacylglycerols than controls.

**Table 1:** Cohort characteristics

	Healthy controls	Anorexia nervosa	*p*
*N* (%)	29 (22%)	102 (78%)	
Chronological age, years	14.0 ± 2.7	14.9 ± 2.0	0.089
Height, cm	159.7 ± 12.2	164.0 ± 8.8	0.082
Height SDS	0.46 ± 0.95	0.26 ± 1.07	0.360
Body weight, kg	52.1 ± 10.1	41.7 ± 8.31	** < 0.001**
Body weight SDS	0.53 ± 0.80	− 0.98 ± 0.97	** < 0.001**
Body mass index, kg/m^2^	20.3 ± 2.4	15.5 ± 2.5	** < 0.001**
Body mass index SDS	0.29 ± 1.00	− 1.78 ± 1.02	** < 0.001**
Creatinine, µmol/L	72 ± 14	67 ± 12	**0.046**
Cystatin C, mg/L	0.76 ± 0.11	0.72 ± 0.15	0.194
eGFR, mL/min/1.73 m^2^	94 ± 17	100 ± 21	0.132
Albumin, g/L	50 ± 5	47 ± 4	**0.001**
Fasting plasma glucose, mmol/L	4.5 ± 0.9	4.3 ± 0.6	0.170
Cholesterol, mmol/L	4.4 ± 0.7	4.5 ± 1.0	0.437
Triacylglycerols, mmol/L	0.7 ± 0.2	0.9 ± 0.4	**0.001**
Uric acid, mmol/L	267 ± 58	227 ± 51	**0.001**
C-reactive protein, mg/L	0.2 (0.1; 0.5)	0.1 (0.1; 0.5)	0.153

Significant differences are given in bold.

*Abbreviations*: *SDS* standard deviation score, *eGFR* estimated glomerular filtration rate.

### Plasma AGE and sRAGE levels

**Fig. 1:**
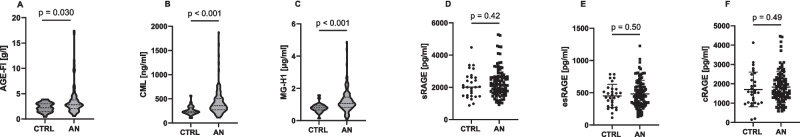
Plasma levels of advanced glycation end products—N^ε^-(carboxymethyllysine), methylglyoxal-derived hydroimidazolone-1 – and soluble receptors for advanced glycation end products in controls and patients with anorexia nervosa. **A** Advanced glycation end-product-associated fluorescence of plasma (AGE-Fl); **B** CML, N^ε^-(carboxymethyl-lysine); **C** MG-H1, methylglyoxal-derived hydroimidazolone-1; **D** sRAGE, the total pool of soluble receptors for advanced glycation end-products; **E** esRAGE, endogenous secretory RAGE; **F** cRAGE, cleaved RAGE, calculated as sRAGE minus esRAGE; CTRL, healthy controls; AN, patients with anorexia nervosa; skewed data (panels **A**, **B**, **C**) are depicted as violin plots with median and interquartile range; normally distributed data (panels C, D, E) are displayed as dot-plots with mean ± SD

Levels of AGE-Fl (Fig. 1A), CML (Fig. 1B), and MG-H1 (Fig. 1C) were significantly higher in girls with AN than in controls. At the same time, both groups displayed similar sRAGE, esRAGE, and cRAGE concentrations (Fig. 1D, E, and F, respectively).

### Girls with anorexia nervosa

#### Bone mineral status and body composition

Data on BMC and BMD in girls with AN are given in Table 2.

**Table 2:** Bone mineral status in girls with anorexia nervosa

	BMC, g	BMD, g/cm^2^	BMD, *z*-score	BMD *z*-score classification, *n* (%)			*P*_Chi_
				> − 1SD	− 1 SD to − 2 SD	≤ − 2 SD	
Lumbar spine	48.8 ± 11.8	0.89 ± 0.14	− 0.38 ± 1.12	65 (63.8)	29 (28.4)	8 (7.8)	0.667
Total hip	28.3 ± 6.6	0.86 ± 0.14	− 0.48 ± 1.14	60 (58.8)	36 (35.3)	6 (5.9)	
Femoral neck	3.8 ± 0.9	0.79 ± 0.13	− 0.47 ± 1.12	59 (57.8)	38 (37.3)	5 (4.9)	

Abbreviations: *BMD* bone mineral density, *SD* standard deviation, *BMC* bone mineral content

The TBS of lumbar vertebrae averaged 1.41 ± 0.08 (range 1.10 to 1.66). Seven girls (6.9%) displayed TBS *z*-scores between − 1 SD and − 2 SD. The prevalence of BMD and TBS scores according to locations is given in the Supplementary file, including the frequency of scores by menstrual status categories (Supplementary Table S2).

Lean and fat mass averaged 32.1 ± 5.9 kg and 9.5 (7.4; 13.0) kg, respectively.

#### Markers of bone turnover

In girls with AN, concentrations of bone formation markers PINP reached 69 (43, 139) ng/mL and that of osteocalcin 25 (15, 39) ng/m; the concentration of bone resorption marker CTX averaged 1.44 ± 1.78 ng/mL. The activity of alkaline phosphatase ranged 1.09 (0.86; 1.49) µkat/L; serum calcium and phosphate levels were 2.43 ± 0.11 mmol/L and 1.31 ± 0.19 mmol/L, respectively; concentration of 25-OH vitamin D reached 33.1 ± 11.0 ng/mL, parathormone averaged 32.6 ± 12.5 ng/L, and IGF-1 levels reached 136 (96; 204) µg/L.

#### Estradiol levels and the trends in bone status markers across menstrual categories

Estradiol levels reached 11.4 (10.0, 24.0) ng/L and were higher in menstruating girls than in premenstrual or those with secondary amenorrhea (Supplementary Table S3). Compared with premenstrual girls, those with primary and secondary amenorrhea had higher TBS, and patients with secondary amenorrhea displayed lower PINP and osteocalcin levels. The ALPK activity was significantly higher in premenstrual girls than in other groups. No significant trend across menstrual status categories was observed for AGEs, sRAGEs, BMD, Ca, 25-OH vitamin D, PTH, and IGF-1 (Supplementary Table S3).

#### Simple correlations

The correlation matrix is given in Supplementary Table S4. Briefly, the BMD of the lumbar spine, hip, and femoral neck showed strong intercorrelations (*r:* 0.81 to 0.91); their relationships with TBS were weaker (*r:* 0.61 to 0.69) but significant. Markers of bone turnover displayed a significant interrelationship (*ρ:* 0.29 to 0.86). Lumbar spine BMD *z*-scores inversely correlated with PINP (*ρ:* − 0.32) and osteocalcin (*ρ:* − 0.28), that of the hip with CTX (*ρ:* − 0.25), and lumbar spine TBS correlated with all three markers of bone turnover (*ρ:* −0.31 to −0.25). AGE-Fl correlated with CTX (*ρ:* − 0.36). EsRAGE displayed inverse associations with markers of bone mineral status (*ρ:* − 0.21 to − 0.25) except for the lumbar spine. CRAGE correlated directly with esRAGE (*ρ:* 0.33), PINP (*ρ:* 0.24), phosphates (*ρ:* 0.24), estradiol (*ρ: *0.24), and inversely with TBS (*ρ:* − 0.21) and MG-H1 (*ρ:* − 0.27).

#### Multiple statistical analyses

The PCA revealed no major outliers; all scores except four were within the 95% Hotelling’s T2 tolerance ellipse (Supplementary Fig. S1).

**Fig. 2:**
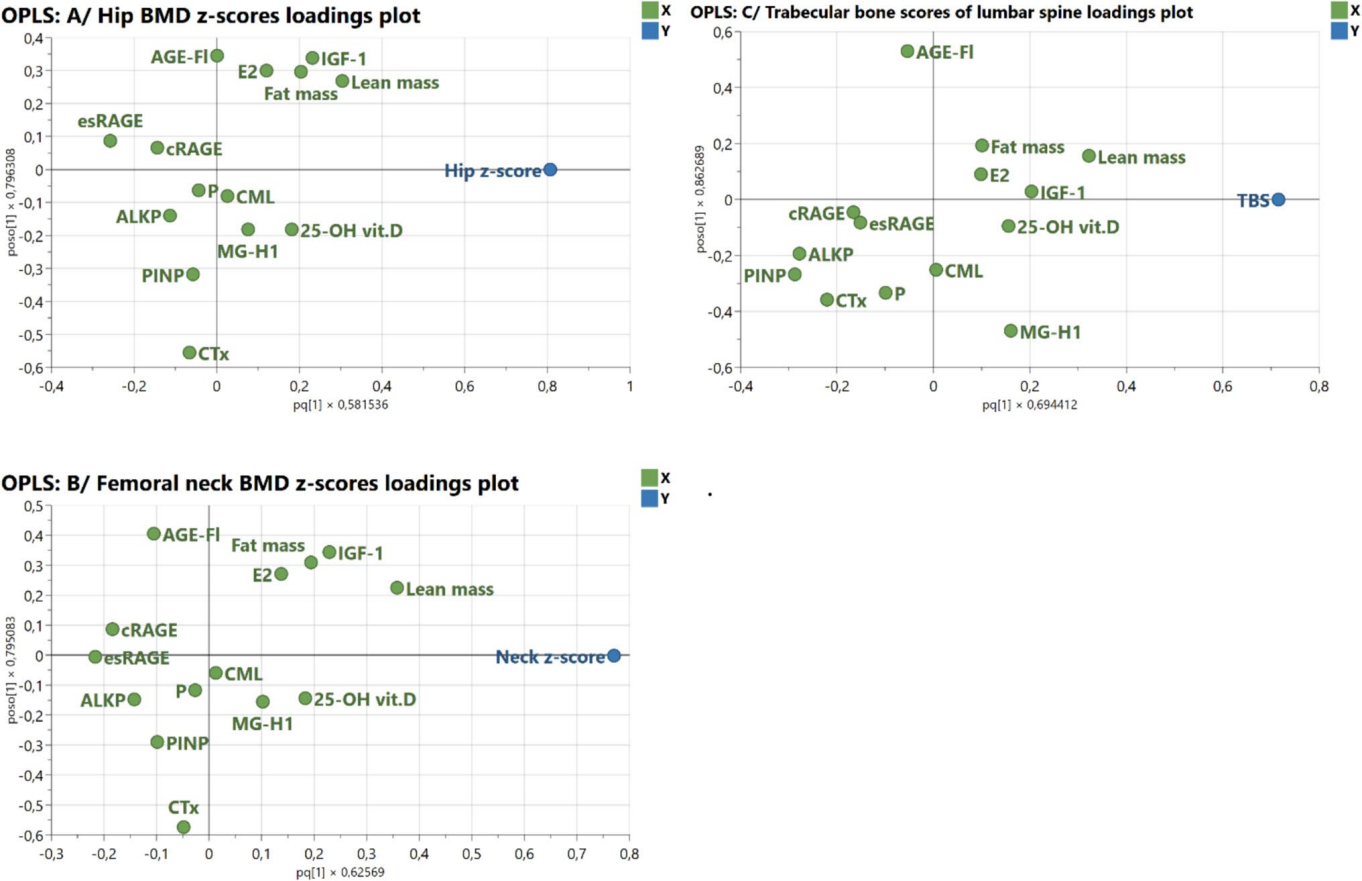
Loading plots visualizing independent predictors of bone mineral density (BMD) *z*-score and trabecular bone (TBS) score using the orthogonal projections to latent structures (OPLS) model in girls with anorexia nervosa. **A** The OPLS model of the hip BMD *z*-score. Independent *x*-variables (in green) adjacent (e.g., lean body mass, fat mass, IGF-1) to the dependent one (hip bone mineral density z-score, in blue) show a positive association with the dependent variable. In contrast, the distant ones (e.g., esRAGE, CTX) are inversely correlated to the dependent one. Significant predictors of the hip BMD *z*-score are given in Table 3; **B** the OPLS model of the femoral neck BMD *z*-score. Independent *x*-variables (in green) adjacent to the dependent one (hip bone mineral density *z*-score, in blue) show a positive association with the dependent variable. In contrast, the distant ones are inversely correlated to the dependent variable. Significant predictors of the femoral neck BMD *z*-score are given in Table 3. **C** The OPLS model of the trabecular bone score of lumbar vertebrae (TBS). Independent *x*-variables (in green) adjacent to the dependent one show a positive association with the dependent variable. In contrast, the distant ones are inversely correlated to the dependent variable. Significant predictors of the TBS are given in Table 3; AGEs, advanced glycation end products-associated fluorescence of plasma; ALKP, alkaline phosphatase; CML, N^ε^-(carboxymethyl)lysine; cRAGE, cleaved receptor for advanced glycation end-products; CTX, carboxy-terminal telopeptide of type I collagen; E2, estradiol; esRAGE, the endogenous secretory receptor for advanced glycation end-products; IGF-1, insulin-like growth factor-1; MG-H1, methylglyoxal-derived hydroimidazolone; P, phosphates; PINP, amino-terminal propeptide of human procollagen type I; 25-OH vit. D, 25-hydroxy vitamin D

The OPLS model selected lean mass, IGF-1, esRAGE, fat mass, CTX, and 25-OH vitamin D as independent predictors of hip BMD *z*-scores (Fig. 2A). The same markers except 25-OH vitamin D predicted femoral neck BMD *z*-scores (Fig. 2B). Lean mass, PINP, ALKP, CTX, and MG-H1 significantly impacted the TBS (Fig. 2C). The VIP values are given in Table 3. The predictive components explained 31–48% of the variation of dependent variables (*R*^2^), but the predictivity of the models was low (*Q*^2^) (Table 3). The permutation plots indicated that the models are valid (Supplementary Fig. S2 A, B, and C, respectively). The model predicting lumbar spine BMD *z*-score was spurious and failed the permutation test validation.

**Table 3:** The multivariate regression of independent variables (predictors) on hip and femoral neck bone mineral density *z*-score and lumbar spine (L2L4) trabecular bone score expressed as variables important for the projection (VIPs) using the orthogonal projections to latent structures (OPLS) model

	Bone mineral density *z*-score		Trabecular bone score
Location	Hip	Femoral neck	Lumbar spine (L1–L4)
Lean mass	**1.74**	**1.86**	**1.55**
IGF-1	**1.42**	**1.33**	0.97
esRAGE	**1.42**	**1.10**	0.73
Fat mass	**1.25**	**1.14**	0.59
CTX	**1.08**	**1.10**	**1.22**
25-OH vit. D	**1.06**	0.97	0.76
Estradiol	0.86	0.86	0.44
cRAGE	0.81	0.94	0.78
ALKP	0.67	0.77	**1.35**
PINP	0.66	0.74	**1.43**
AGE-Fl	0.63	0.92	0.97
MG-H1	0.53	0.60	**1.13**
Phosphates	0.26	0.26	0.75
CML	0.21	0.13	0.45
*R*^2^	0.31	0.39	0.48
*Q*^2^	0.07	0.19	0.39

VIP values ≥ 1 are considered as significant (given in bold)

*Abbreviations*: *L1–L4* lumbar spine vertebrae 1 to 4, *IGF-1* insulin-like growth factor-1, *esRAGE* the endogenous secretory receptor for advanced glycation end-products, *CTX* carboxy-terminal telopeptide of type I collagen, *25-OH vit. D* 25-hydroxy vitamin D, *cRAGE* cleaved receptor for advanced glycation end-products, *ALKP* alkaline phosphatase, *PINP* amino-terminal propeptide of human procollagen type I, *AGE-Fl* advanced glycation end-products-associated fluorescence of plasma, *MG-H1* methylglyoxal-derived hydroimidazolone, *CML* N^ε^-(carboxymethyl)lysine, *R*^*2*^ variance, *Q*^*2*^ predictability

## Discussion

Our former studies showed that girls with AN display higher AGE-associated fluorescence of plasma than controls [19, 21]. Herein, we aimed to confirm that elevated AGE-Fl reflects the accumulation of chemically defined AGEs. We show that, at the time of diagnosis, girls with AN, except for higher AGE-Fl, also present with higher plasma levels of CML and MG-H1 than their healthy peers. Lacking intercorrelation between the AGE levels might reflect the potential interference of unknown fluorescence compounds with AGE-Fl determination [22], the fact that CML and MG-H1 lack intrinsic fluorescence, and that the formation pathways of bulk AGEs, CML, and MG-H1 differ [1, 2]. In contrast to our hypothesis, AGEs did not predict bone mineral status in patients with AN. Although soluble RAGE variants showed similar levels between the groups, our data suggests that esRAGE might predict bone mineral status in girls with AN.

The higher MG-H1 levels in girls with AN may result from increased MGO production and/or reduced degradation. Methylglyoxal, the most potent glycating agent, modifies arginine residues to form MG-H1 [1]. MGO primarily originates from glucose metabolism, but alternative sources such as ketone bodies, amino acids, or lipid peroxidation products contribute more significantly to MGO formation during starvation-induced catabolism. Since MGO is highly toxic, it is detoxified by a glutathione-dependent glyoxalase system into D-lactate [1]. Lower plasma glutathione levels in patients with AN than in healthy controls [19, 29] suggest reduced glyoxalase activity, potentially leading to MG-H1 accumulation. Determination of glyoxalase activity or D-lactate as a surrogate marker is needed to confirm this hypothesis.

CML is formed via non-enzymatic glycation, glycoxidation, and lipid peroxidation reactions [2]. AN is associated with oxidative stress: Prolonged starvation and physical and psychological stress induce the production of ROS, which are not efficiently neutralized by antioxidant systems due to insufficient vitamin and micronutrient intake [18–20]. Similar to our study, young women with reduced bone mass and adults with osteoporosis displayed higher plasma CML, bulk AGEs, or pentosidine levels than healthy controls [23, 30, 31].

The cross-sectional design of our study limits conclusions about the mechanisms behind elevated plasma AGE levels in girls with AN. These AGEs are of endogenous origin: Only low molecular weight dietary AGEs are absorbed into the circulation, and these do not impact plasma levels of protein-bound AGEs [32], quantified in our study. Chronic hyperglycemia can be ruled out as a cause of the exaggerated formation of AGEs. Normal kidney function excludes renal involvement in the accumulation of plasma AGEs [33]. We propose that oxidative stress, due to antioxidant deficiency, may drive AGE overproduction in patients with AN. Circulating CML levels are higher in AN than in healthy controls. The opposite occurs in obesity [34–36], where low plasma CML is linked to its deposition in adipose tissue via a RAGE-dependent mechanism [35, 36]. In adults, total fat mass inversely correlated with CML [36], but the relationship was positive in our patients. While protein-bound MG-H1 levels were similar in obese and lean subjects, MGO and D-lactate levels were higher in obese [37, 38]. This may be due to MGO accumulation in fat tissue and a plasma-to-interstitial shift of albumin in obesity [38]. These contrasting AGE patterns in patients with AN and obesity suggest that a low volume of adipose tissue may limit AGE deposition, leading to higher circulating AGE levels in patients with AN.

Decreased BMC and BMD of cortical and trabecular bone are early complications of AN [15, 39]. Alarmingly, at diagnosis, fewer than half of the young girls with AN had healthy bones (BMD *z*-scores > − 1SD at all three measured sites), and among those with *z*-scores ≤ − 1 SD, 21% scored ≤ − 2 SD at least at one site, consistent with previous reports [40, 41]. In our patients, deterioration of BMD occurred before a decrease in TBS. Our former study suggested a link between low BMD and high plasma AGE levels as AGE-Fl inversely correlated with total hip BMD *z*-score when girls with AN and controls were evaluated together. However, our study lacks data on bone mineral status for controls, and no significant correlations between AGEs and BMD or TBS were revealed in girls with AN, nor did the multivariate model select plasma AGEs as significant determinants of bone mineral status. In adults without diabetes, plasma pentosidine (a cross-linking AGE) levels correlated positively with its bone content, and both correlated positively with the osteoporosis severity [42]. Similar data for CML or MG-H1 relationship in plasma and bone is lacking.

Evidence linking plasma AGEs to bone mineral status in subjects without diabetes is primarily derived from elderly subjects and is limited or inconsistent. In postmenopausal women or patients with osteoporosis, BMD and TBS negatively correlated with bulk AGEs and CML [23, 31, 43]. However, large-scale studies reported no significant association between plasma CML or pentosidine and BMD [44, 45]. The etiopathogenesis of impaired bone mass in postmenopausal women and elderly populations differs from that in AN, making direct comparisons between our findings and those mentioned challenging. Aging-related osteoporosis is influenced by factors such as low physical activity, impaired calcium absorption, lower vitamin D levels, comorbidities, medication use, and, in females, a natural, gradual decline in estrogen levels due to ovarian failure. In contrast, low bone mass in patients with AN is driven by hormonal imbalance caused by hypothalamic-pituitary axis dysfunction due to nutritional deficiencies, alongside lean and fat mass reduction, even in patients who engage in vigorous physical activity [39].

Since RAGE expression is typically low under homeostatic conditions and upregulated by AGEs [3], we anticipated that generating sRAGE production through shedding RAGE from the cell surface or alternative splicing would be enhanced. RAGE functions as a multiligand pattern recognition receptor, binding several ligands except for protein-bound AGEs, including those involved in bone remodeling or anorexia pathophysiology, such as amyloid-β, S100 calcium-binding proteins, or high mobility group box 1 proteins [46, 47]. However, our patients exhibited sRAGE variant levels comparable to the controls. Thus, contrary to our hypothesis, this suggests that sRAGE levels may not reflect overall sRAGE production reliably. In our girls with AN, cRAGE inversely correlated with MG-H1. A negative relationship between soluble RAGEs and their ligands aligns with the idea that sRAGEs act as decoy receptors, exerting biological effects [5]. While no significant differences in sRAGE levels were observed between the patients and controls, the potential scavenging role of cRAGE in AN cannot be ruled out. Additionally, esRAGE levels were inversely correlated with markers of bone mineral status, consistent with previous findings in females with rheumatoid arthritis [48]. Moreover, high sRAGE levels have been proposed to inhibit RANKL-induced osteoclastogenesis [49].

Estrogen deficiency is associated with reduced BMD [41, 50]. While we observed weak correlations between estradiol and certain markers of BMD, bone turnover and metabolism, AGEs, and cRAGE, the multivariate model did not identify estradiol as a significant predictor of BMD *z*-score or TBS in girls with AN. This discrepancy is likely due to our patients’ heterogeneity in the menstrual status. To date, the relationships between hypothalamic-pituitary axis hormones and AGE or sRAGE levels—whether in healthy individuals or AN-related hypothalamic-pituitary dysfunction—remain unexplored.

The main limitation of our study is its cross-sectional design, along with the absence of DEXA measurements and data on markers of bone turnover and estradiol in the healthy controls. However, to this point, our findings can be compared to those from our pilot study [21]. On the other hand, we provide the first data on the plasma levels of chemically defined protein-bound AGEs and sRAGE variants and their relationship with bone mineral status in a reasonably large cohort of girls with AN. We also show that in AN, predictors of TBS differ from those of BMD and that deterioration of BMD occurs before a decline in TBS.

## Conclusions

Available data unequivocally indicates that plasma AGE levels are higher in subjects with low bone mass—including our girls with AN and adults with osteopenia or osteoporosis [23, 30, 31])—than in healthy controls. Yet the causality of this association remains uncertain. Our findings indicate that higher levels of non-crosslinking plasma AGEs are unlikely to directly impact bone mineral status and turnover in young girls with AN. This contradicts findings in postmenopausal women with osteopenia or osteoporosis [23, 31, 43], suggesting that underlying mechanisms of bone impairment differ between these populations. Still, bone quality might be determined by cross-linking AGEs, such as pentosidine [30, 42], which were not analyzed in our study.

Our data foster discussion on whether high esRAGE levels protect from inflammation, oxidative stress, and related pathologies or instead reflect the overstimulation of cell surface RAGE leading to the aggravation of pathological conditions [4]. An important question remains whether similar associations between AGEs, sRAGEs, and bone mineral status or markers of bone turnover exist in healthy individuals, patients with the binge eating/purging type of AN, or males with AN.

Addressing the underlying eating disorder is crucial for improving bone health in AN. Weight restoration through re-alimentation improves oxidative balance [20], increases fat mass, and effectively, albeit slowly, promotes BMD increase [51]. However, the impact of this process on the AGEs/RAGE axis remains unclear. Future research should explore the interaction between oxidative status, AGEs/RAGE axis, bone health, and the potential regulatory role of fat mass in patients with AN. Longitudinal studies are warranted to elucidate whether high esRAGE levels are causally linked to low bone mass or merely represent an epiphenomenon. Investigating the dynamics of sRAGE over time could provide valuable insights into its role as a potential biomarker for monitoring bone health and disease progression in AN. Moreover, the potential pathogenetic role of elevated AGEs in other AN-associated medical complications, such as cardiovascular (including echocardiographic findings), myocardial and brain atrophy, low blood cell counts, multiple endocrine abnormalities, and depression [15], should be investigated. A question arises whether integrating monitoring of the AGEs/sRAGE axis into clinical practice would offer a novel approach for assessing disease status and guiding personalized interventions to mitigate long-term health consequences in patients with AN.

## Supplementary Information

Below is the link to the electronic supplementary material.

Supplementary file1 (DOCX 747 KB)

## Data Availability

Data are available on reasonable request from the corresponding author.

## Declarations

### Disclaimer

The funders had no role in the study design, data analysis, publication decision, or manuscript preparation.
