# Supplementary material for: Association of plasma levels of protein-bound advanced glycation end-products and their soluble receptors with bone mineral status in young girls with the restrictive type of anorexia nervosa
Source: Arch Osteoporos. 2025 Aug 27;20(1):116. doi: 10.1007/s11657-025-01554-z (PMC12391243; doi:10.1007/s11657-025-01554-z)
Supplement: Supplementary file 1 — Supplementary file1 (DOCX 747 KB) [file 11657_2025_1554_MOESM1_ESM.docx]

Supplementary material to:

**Association of plasma levels of protein-bound advanced glycation end-products and their soluble receptors with bone mineral status in young girls with the restrictive type of anorexia nervosa**

Katarína Šebeková^1^, Alexandra Gaál Kovalčíková^2^, Alžbeta Čagalová^2^, Ľubica Tichá^2^, Ľudmila Podracká^2^

^1^Institute of Molecular Biomedicine, Faculty of Medicine, Comenius University, Sasinkova 4, 811 08 Bratislava, Slovakia

^2^Department of Pediatrics, Faculty of Medicine, National Institute of Children's Diseases, Comenius University, Limbova 1, 833 40 Bratislava, Slovakia

Correspondence: Katarína Šebeková, Institute of Molecular Biomedicine, Faculty of Medicine, Comenius University, Sasinkova 4, 811 08 Bratislava, Slovakia; email: [sebekova3@uniba.sk](mailto:sebekova3@uniba.sk); [kata.sebekova@gmail.com](mailto:kata.sebekova@gmail.com)

**Diagnostic procedures**

The diagnosis was based on parental information, patient observation, clinical examination and interviews, and consular evaluations by a psychiatrist or psychologist to exclude other psychiatric disorders. Restrictive type of AN was diagnosed on the following criteria: persistent energy intake restriction leading to significantly low body weight, intense fear of gaining weight or becoming fat or persistent behavior that interferes with weight gain, disturbance in self-perceived body weight or shape, and a lack of recurrent episodes of binge eating or purging behavior in the previous three months. Thus, weight loss was achieved through dieting, fasting, and, in some cases, excessive exercise.

Secondary amenorrhea was defined as an absence of regular menstruation for at least 3 months with previously normal menstruation, and primary amenorrhea as a failure of menarche by the age of 15 years [1].

**Methods**

**DEXA:** The DEXA scanner (Horizon QDR instrument, Hologic Inc., Danbury, CT, USA) was calibrated daily using a standard block supplied by the manufacturer. Girls were examined in the hospital clothing after removing all metal items.

**Laboratory procedures:** Blood collected into K_3_EDTA, lithium-heparin, and serum tubes with gel & clot activator (Vacutainer Plastic Tube, Becton Dickinson, Czech Republic) was centrifuged.

**Routine blood chemistry** was performed using the Cobas c501 analyzer (Roche Diagnostic, Mannheim, Germany).

**25-OH vitamin D** levels were determined using the chemiluminescent method (Vitros 5600 analyzer, Ortho Clinical Diagnostic, Raritan, NJ, USA).

**Parathormone, osteocalcin,** **PINP,** **carboxy-terminal telopeptide of type I collagen (CTX), estradiol, and insulin-like growth factor-1 (IGF-1)** levels were quantified using the electrochemiluminescence method (Cobas e411, Roche Diagnostic, Mannheim, Germany).

**Cystatin C** concentration was quantified immunoturbidimetrically (Biolis 24i Premium analyzer, Tokyo Boeki Machinery, Tokyo, Japan)

**ELISA methods**, according to the manufacturer's instructions, were used to quantify concentrations of plasma **CML and MG-H1** (both: Cell Biolabs, Inc., San Diego, CA, USA), **sRAGE** (R&D Systems Inc., Minneapolis, MN, USA, which determines the total pool of all soluble forms of RAGE, including esRAGE), and **esRAGE** (manufactured by Daiichi Fine Chemicals Co. Ltd., Takaoka, Japan and distributed by BBridge International, Inc.; Cupertino, USA).

**AGE-associated fluorescence of plasma (AGE-Fl)** was determined as described previously [2, 3]

**Spectrophotometric and fluorometric measurements** were performed on a Synergy HT Multi-Mode Microplate Reader (BioTekInstruments, Inc., Winooski, VT, USA).

**Statistical analyses:** Non-parametric tests were applied if at least one data set was skewed. Before multivariate modeling, the multicollinearity of independent variables was tested by determining a variance inflation factor (VIF, Supplementary Table S1).

**Supplementary Table S1:** Variance inflation factors (VIF) calculated for multivariate regression models

|  | VIF | |
| --- | --- | --- |
| esRAGE | 1.52 | 1.41 |
| cRAGE | 1.93 | 1.91 |
| Alkaline phosphatase | 2.40 | 2.06 |
| Insulin-like growth factor-1 | 2.38 | 2.38 |
| 25-OH vitamin D | 1.43 | 1.28 |
| Phosphates | 1.36 | 1.31 |
| PINP | 9.43 | 1.67 |
| Estradiol | 1.52 | 1.52 |
| MG-H1 | 1.32 | 1.26 |
| CML | 1.62 | 1.59 |
| CTX | 1.62 | 1.60 |
| Osteocalcin | 12.8 | -- |
| Lean mass | 1.92 | 1.84 |
| Fat mass | 2.08 | 1.95 |
| AGE-Fl | 1.43 | 1.42 |

esRAGE, endogenous secretory receptor for advanced glycation end-products; cRAGE cleaved receptor for advanced glycation end-products; PINP, amino-terminal propeptide of human procollagen type I; eGFR, estimated glomerular filtration rate; MG-H1, methylglyoxal-derived hydroimidazolone; CML, N^ε^-(carboxymethyl)lysine; CTX, carboxy-terminal telopeptide of type I collagen; AGE-Fl, advanced glycation end-products-associated fluorescence of plasma

**Multivariate analysis** (PCA, OPLS): Variables with high skewness and low min-to-max ratios were logarithmically transformed, and data were standardized to z-scores. The OPLS models were validated using the permutation test with 100 random permutations to exclude potential overfitting. The criterion for validity was that all Q^2^ values to the left in the plot were lower than the original point to the right and that the regression line of the Q^2^ intersected the vertical axis at or below zero.

**Supplementary Table S2:** Bone mineral density z-score in patients with anorexia according to the menstrual status

| **BMD z-score** | **Premenstrual (n=24)** | **Primary amenorrhea (n=7)** | **Secondary amenorrhea (n=61)** | **Menstruating (n=10)** | **Total** |
| --- | --- | --- | --- | --- | --- |
| **> -1SD** | 4 (16.7%) | 3 (42.8%) | 33 (54.1%) | 5 (50%) | 45 |
| **-1 SD to -2 SD** | 19 (79.2%) | 2 (28.6%) | 22 (36.1%) | 2 (20%) | 45 |
| **≤ -2 SD** | 1 (4.1%) | 2 (28.6%) | 6 (9.8%) | 3 (30%) | 12 |
| Total | 24 (100%) | 7 (100%) | 61 (100%) | 10 (100%) | 102 |

BMD, bone mineral density; SD, standard deviation

Among 102 girls with AN, forty-five (44.1%) presented with BMD z-scores between -1 SD and -2 SD: thirteen at a single location, twenty at two sites, and twelve at all three regions. Twelve patients (11.8%) had at least one BMD z-score ≤ -2 SD: eight at a single location, one at two sites, and three girls showed BMD z-scores ≤ -2 SD at all three regions. Eighty-three percent of premenstrual girls, 57% with primary amenorrhea, 46% suffering from secondary amenorrhea, and 50% of menstruating patients presented with BMD z-score < -1SD (Supplementary Table S2).

The trabecular bone score of lumbar vertebrae averaged 1.41±0.08 (range: 1.10 to 1.66). Ninety-five (93.1%) patients presented with TBS z-score > -1SD, and 7 girls (6.9%) displayed TBS z-scores between -1 SD and -2 SD. One of them was premenstrual, four presented with secondary amenorrhea, and two girls reported that they were menstruating. Two patients with TBS z-scores between -1 SD and -2 SD also displayed BMD z-scores between -1 SD and -2 SD (at two and three regions, respectively), and the remaining five had at least one BMD z-score ≤ -2 SD (three of them at all three loci).

**Supplementary Table S3:** Plasma concentrations of estradiol, soluble receptors for advanced glycation end-products, and advanced glycation end-products in girls with anorexia nervosa according to menstrual status

|  | **Premenstrual (n=24)** | **Primary amenorrhea (n=7)** | **Secondary amenorrhea (n=61)** | **Menstruating (n=10)** | **p** |
| --- | --- | --- | --- | --- | --- |
| Estradiol (ng/l) | 10 (10; 13)^*^ | 10 (10; 34) | 12 (10; 23)^*^ | 31 (20; 89) | **0.004** |
| sRAGE (pg/ml) | 2356±990 | 2354±795 | 2253±858 | 2516±1010 | 0.838 |
| esRAGE (pg/ml) | 497±200 | 465±114 | 484±229 | 436±187 | 0.902 |
| cRAGE (pg/ml) | 1860±883 | 1889±771 | 1769±766 | 2081±931 | 0.734 |
| AGE-Fl (g/l) | 2.8±1.1 | 2.3±1.2 | 3.7±2.9 | 4.8±4.9 | 0.163 |
| CML (ng/ml) | 411±301 | 384±145 | 436±247 | 576±492 | 0.433 |
| MGH-1 (µg/ml) | 1.2±0.5 | 1.7±1.5 | 1.2±0.7 | 1.2±0.7 | 0.352 |
| LS BMD z-score | -0.69±0.81 | -0.79±1.18 | -0.18±1.15 | -0.61±1.39 | 0.156 |
| T. hip BMD z-score | -0.90±0.86 | -0.67±0.82 | -0.26±1.83 | -0.63±1.66 | 0.112 |
| F. neck BMD z-score | -0.91±0.80 | -0.70±1.05 | -0.25±1.12 | -0.63±1.53 | 0.081 |
| TBS lumbar vtb. | 1.36±0.09 | 1.45±0.06^+^ | 1.42±0.06^++^ | 1.43±0.10 | **0.004** |
| PINP (ng/ml) | 250±234 | 245±439 | 72±59^++^ | 150±145 | **0.001** |
| Osteocalcin (ng/ml) | 61±51 | 49±60 | 23±18^+++^ | 42±26 | **<0.001** |
| CTX (ng/ml) | 1.7±0.7 | 1.9±1.1 | 1.4±0.8 | 1.0±0.5 | **0.033^a^** |
| ALKP (µkat/l) | 2.1±1.0 | 1.0±0.5^++^ | 1.0±0.4^+++^ | 1.2±0.5^++^ | **<0.001** |
| Ca (mmol/l) | 2.44±0.08 | 2.44±0.11 | 2.43±0.12 | 2.42±0.12 | 0.929 |
| P (mmol/l) | 1.40±0.20 | 1.20±0.32 | 1.29±0.17 | 1.36±0.13 | **0.032^a^** |
| 25-OH vit. D (ng/ml) | 30±11 | 35±6 | 34±11 | 30±14 | 0.407 |
| PTH (ng/ml) | 32±13 | 28±12 | 32±12 | 41±15 | 0.099 |
| IGF-1 (µg/l) | 139±105 | 132±70 | 182±109 | 237±168 | 0.129 |

sRAGE, soluble receptor for advanced glycation end-products; esRAGE, endogenous secretory receptor for advanced glycation end-products; cRAGE leaved receptor for advanced glycation end-products; AGE-Fl, advanced glycation end-products-associated fluorescence of plasma; CML, N^ε^-(carboxymethyl)lysine; MG-H1, methylglyoxal-derived hydroimidazolone; LS, lumbar spine BMD, bone mineral density; T, total; F, femoral; TBS, trabecular bone score; vtb., vertebrae; PINP, amino-terminal propeptide of human procollagen type I; CTX, carboxy-terminal telopeptide of type I collagen; ALKP, alkaline phosphatase; Ca, calcium; P, phosphates; 25-OH vit. D, 25-hydroxy vitamin D; PTH, parathormone; IGF-1, insulin-like growth factor-1; data given as median (interquartile range) were compared using the Kruskal-Wallis with post-hoc Dunns` tests; normally distributed data were compared using the ANOVA with post-hoc Bonferroni correction and are given as mean±SD; *, p<0.05 vs. menstruating girls; +: p<0.05, ++: p<0.01, +++: p<0.001 vs. premenstrual girls; a: the post-hoc test failed to localize significance

**Supplementary Table S4:** Correlations between variables in girls with anorexia nervosa

|  | Hip | Neck | TBLS | CTX | *PINP* | *Osteo* | *CML* | *MGH1* | *AGE-Fl* | esR | cR | *ALKP* | 25OHvD | PTH | Ca | P | eGFR | *CRP* | *IGF1* | LM | FM | *E2* |
| --- | --- | --- | --- | --- | --- | --- | --- | --- | --- | --- | --- | --- | --- | --- | --- | --- | --- | --- | --- | --- | --- | --- |
| Lumb | **0.812**  **<0.001** | **0.828**  **<0.001** | **0.691**  **<0.001** | -0.205  0.054 | ***-0.317***  ***0.003*** | ***-0.281***  ***0.009*** | *0.073*  *0.468* | *0.079*  *0.429* | *-0.005*  *0.960* | -0.164  0.101 | -0.182  0.068 | ***-0.371***  ***<0.001*** | O.182  0.068 | -0.012  0.906 | *-0.134*  *0.163* | -0.145  0.098 | 0.074  0.474 | *0.090*  *0.414* | *0.169*  *0.106* | **0.571**  **<0.001** | **0.200**  **0.044** | *0.180*  *0.074* |
| Hip | -- | **0.907**  **<0.001** | **0.605**  **<0.001** | **-0.254**  **0.015** | *-0.207*  *0.059* | *-0.145*  *0.184* | *0.075*  *0.454* | *0.070*  *0.489* | *-0.015*  *0.881* | **-0.246**  **0.013** | -0.125  0.283 | ***-0.333***  ***<0.001*** | **0.206**  **0.039** | -0.108  0.288 | *0.003*  *0.799* | -0.193  0.056 | 0.120  0.242 | *0.129*  *0.291* | ***0.273***  ***0.008*** | **0.588**  **<0.001** | **0.275**  **0.005** | ***0.220***  ***0.028*** |
| Neck |  | **--** | **0.611**  **<0.001** | -0.205  0.054 | *-0.199*  *0.069* | *-0.187*  *0.085* | *0.038*  *0.703* | *0.138*  *0.170* | *-0.018*  *0.857* | **-0.233**  **0.019** | -0.172  0.085 | ***-0.326***  ***<0.001*** | **0.204**  **0.041** | -0.100  0.326 | *-0.023*  *0.819* | -0.151  0.130 | 0.104  0.309 | *0.097*  *0.382* | ***0.284***  ***0.006*** | **0.638**  **<0.001** | **0.216**  **0.029** | ***0.238***  ***0.017*** |
| TBLS |  |  | -- | **-0.313**  **0.003** | ***-0.264***  ***0.015*** | ***-0.245***  ***0.023*** | *0.082*  *0.414* | *0.177*  *0.082* | *-0.031*  *0.757* | **-0.210**  **0.035** | **-0.214**  **0.032** | ***-0.272***  ***0.006*** | **0.200**  **0.045** | -0.058  0.566 | *-0.067*  *0.506* | -0.136  0.174 | 0.190  0.062 | *-0.057*  *0.878* | ***0.320***  ***0.002*** | **0.436**  **<0.001** | 0.112  0.263 | *0.196*  *0.051* |
| CTX |  |  |  | -- | ***0.294***  ***0.007*** | ***0.320***  ***0.003*** | *0.105*  *0.327* | *0.107*  *0.319* | ***-0.359***  ***0.001*** | 0.109  0.310 | -0.055  0.611 | *0.156*  *0.147* | 0.156  0.147 | -0.114  0.296 | *0.048*  *0.657* | 0.100  0.351 | -0.027  0.809 | ***-0.271***  ***0.016*** | ***-0.342***  ***0.002*** | **0.280**  **0.008** | -0.171  0.110 | ***-0.287***  ***0.007*** |
| *PINP* |  |  |  |  | -- | ***0.861***  ***<0.001*** | *0.010*  *0.930* | *-0.034*  *0.759* | *-0.150*  *0.175* | *0.068*  *0.527* | ***0.238***  ***0.029*** | ***0.693***  ***<0.001*** | *-0.0078*  *0.952* | *-0.012*  *0.913* | *0.058*  *0.598* | ***0.488***  ***<0.001*** | *-0.159*  *0.155* | *0.138*  *0.242* | *0.226*  *0.048* | ***-0.310***  ***0.004*** | *0.185*  *0.092* | ***0.281***  ***0.010*** |
| *Osteo* |  |  |  |  |  | *--* | *0.208*  *0.055* | *-0.100*  *0.362* | *-0.139*  *0.204* | *-0.024*  *0.825* | *0.160*  *0.141* | ***0.707***  ***<0.001*** | *-0.082*  *0.456* | *0.120*  *0.279* | *0.039*  *0.719* | ***0.489***  **<0.001** | *-0.147*  *0.183* | ***0.252***  ***0.030*** | *0.184*  *0.104* | ***-0.200***  ***0.006*** | ***0.314***  ***0.003*** | ***0.359***  ***0.001*** |
| *CML* |  |  |  |  |  |  | *--* | *0.106*  *0.293* | *-0.101*  *0.255* | *0.004*  *0.968* | *0.063*  *0.530* | *0.062*  *0.541* | *0.076*  *0.449* | *0.051*  *0.615* | *0.119*  *0.233* | *0.069*  *0.491* | *0.016*  *0.879* | *0.197*  *0.073* | *-0.048*  *0.649* | *-0.092*  *0.357* | ***0.202***  ***0.041*** | ***0.210***  ***0.036*** |
| *MGH1* |  |  |  |  |  |  |  | *--* | *-0.109*  *0.221* | *-0.101*  *0.318* | ***-0.287***  ***0.004*** | *-0.092*  *0.363* | ***0.228***  ***0.023*** | *0.034*  *0.740* | *-0.120*  *0.231* | *0.031*  *0.755* | *0.070*  *0.500* | *0.082*  *0.459* | *0.073*  *0.492* | *0.050*  *0.621* | *-0.103*  *0.306* | *-0.010*  *0.923* |
| *AGE-Fl* |  |  |  |  |  |  |  |  | *--* | *-0.049*  *0.629* | *0.162*  *0.106* | *-0.093*  *0.358* | *-0.177*  *0.078* | *0.141*  *0.168* | *-0.053*  *0.597* | *0.038*  *0.709* | *-0.151*  *0.142* | *0.134*  *0.227* | *0.191*  *0.069* | *0.126*  *0.210* | *-0.045*  *0.654* | *-0.040*  *0.697* |
| esRAGE |  |  |  |  |  |  |  |  |  | -- | **0.328**  **0.006** | *-0.009*  *0.927* | -0.096  0.343 | 0.003  0.974 | *0.101*  *0.316* | 0.063  0.529 | -0.165  0.106 | *-0.055*  *0.620* | *-0.198*  *0.057* | -0.021  0.838 | -0.180  0.071 | *-0.032*  *0.752* |
| cRAGE |  |  |  |  |  |  |  |  |  |  | -- | *0.134*  *0.187* | -0.182  0.069 | -0.022  0.828 | *0.136*  *0.174* | **0.242**  **0.015** | **-0.344**  **0.001** | *0.193*  *0.079* | *0.121*  *0.239* | -0.061  0.547 | 0.029  0.766 | ***0.235***  ***0.019*** |
| *ALKP* |  |  |  |  |  |  |  |  |  |  |  | *--* | *-0.141*  *0.163* | *0.152*  *0.138* | *0.108*  *0.286* | ***0.361***  ***<0.001*** | *-0.115*  *0.267* | ***0.240***  ***0.030*** | ***0.291***  ***0.009*** | ***-0.323***  ***0.001*** | ***0.314***  ***0.001*** | ***0.297***  ***0.003*** |
| 25OHD |  |  |  |  |  |  |  |  |  |  |  |  | -- | -0.015  0.885 | *0.015*  *0.630* | -0.114  0.256 | **0.264**  **0.009** | *-0.206*  *0.060* | *0.068*  *0.942* | 0.080  0.425 | -0.056  0.578 | *-0.045*  *0.661* |
| PTH |  |  |  |  |  |  |  |  |  |  |  |  |  | -- | *-0.015*  *0.883* | 0.140  0.166 | -0.174  0.094 | ***0.226***  ***0.042*** | *0.038*  *0.727* | 0.109  0.282 | 0.171  0.090 | ***0.336***  ***0.001*** |
| Ca |  |  |  |  |  |  |  |  |  |  |  |  |  |  | -- | *-0.043*  *0.669* | *0.006*  *0.951* | *-0.112*  *0.312* | *0.088*  *0.402* | -0.065  0.519 | 0.123  0.217 | *-0.003*  *0.974* |
| P |  |  |  |  |  |  |  |  |  |  |  |  |  |  |  | -- | -0.141  0.168 | *0.151*  *0.169* | ***0.265***  ***0.010*** | -0.007  0.945 | 0.058  0.565 | ***0.202***  ***0.044*** |
| eGFR |  |  |  |  |  |  |  |  |  |  |  |  |  |  |  |  | -- | *-0.168*  *0.128* | *-0.069*  *0.519* | 0.097  0.343 | 0.077  0.454 | *-0.132*  *0.204* |
| *CRP* |  |  |  |  |  |  |  |  |  |  |  |  |  |  |  |  |  | *--* | *0.215*  *0.060* | *0.101*  *0.360* | ***0.305***  ***0.005*** | *0.196*  *0.078* |
| *IGF-1* |  |  |  |  |  |  |  |  |  |  |  |  |  |  |  |  |  |  | *--* | ***0.435***  ***<0.001*** | ***0.535***  ***<0.001*** | ***0.517***  ***<0.001*** |
| LM |  |  |  |  |  |  |  |  |  |  |  |  |  |  |  |  |  |  |  | -- | **0.254**  **0.010** | ***0.255***  ***0.010*** |
| *FM* |  |  |  |  |  |  |  |  |  |  |  |  |  |  |  |  |  |  |  |  | ***--*** | ***0.498***  ***<0.001*** |

Lumb, lumbar spine bone mineral density z-score; Hip, left hip bone mineral density z-score; Neck, left femoral neck bone mineral density z-score; TBLS, lumbar spine trabecular bone (L2-L4) score; CTX, carboxy-terminal telopeptide of type I collagen; PINP, amino-terminal propeptide of human procollagen type I; Osteo, osteocalcin; CML, N^ε^-(carboxymethyl)lysine; MG-H1, methylglyoxal-derived hydroimidazolone; AGE-Fl, advanced glycation end-products-associated fluorescence of plasma; sRAGE, soluble receptor for advanced glycation end-products; esRAGE, endogenous secretory receptor for advanced glycation end-products; cRAGE leaved receptor for advanced glycation end-products; ALKP, alkaline phosphatase; 25-OHvD, 25-hydroxy vitamin D; PTH, parathormone; Ca, calcium, P, phosphates; eGFR, estimated glomerular filtration rate; CRP, C-reactive protein; IGF-1, insulin-like growth factor-1; LM, lean mass; FM, fat mass; E2, estradiol; Pearson correlations (r, between two sets of normally distributed data) are given in standard fonts, Spearman correlations (ρ, between the sets in which at least one variable did not fit to gaussian distribution) are given in italics; significant correlations (p<0.05) are given in bold

**Supplementary Fig. S1:** The principal component analysis


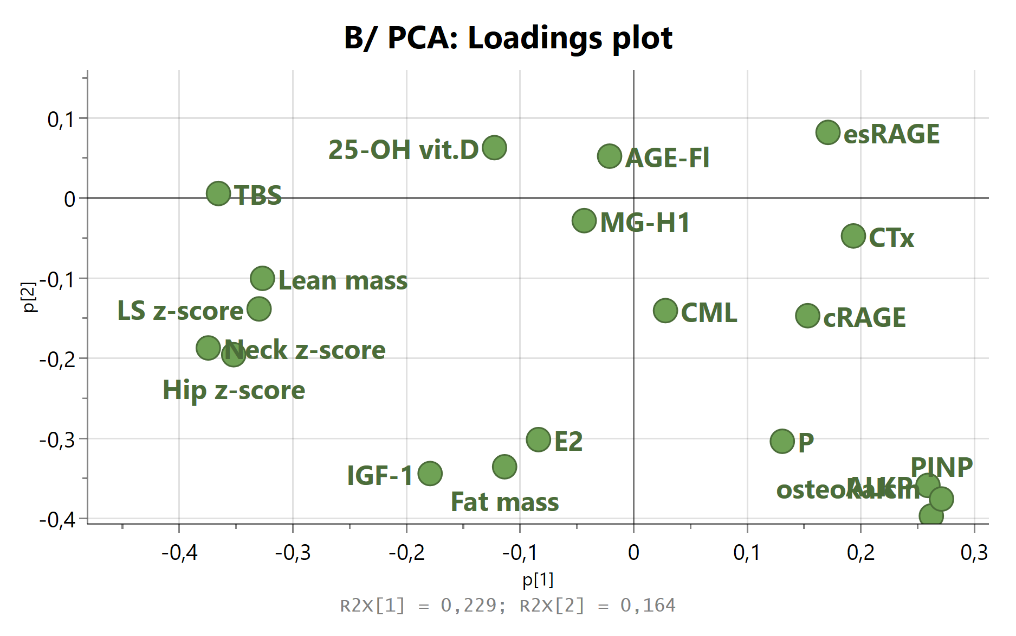

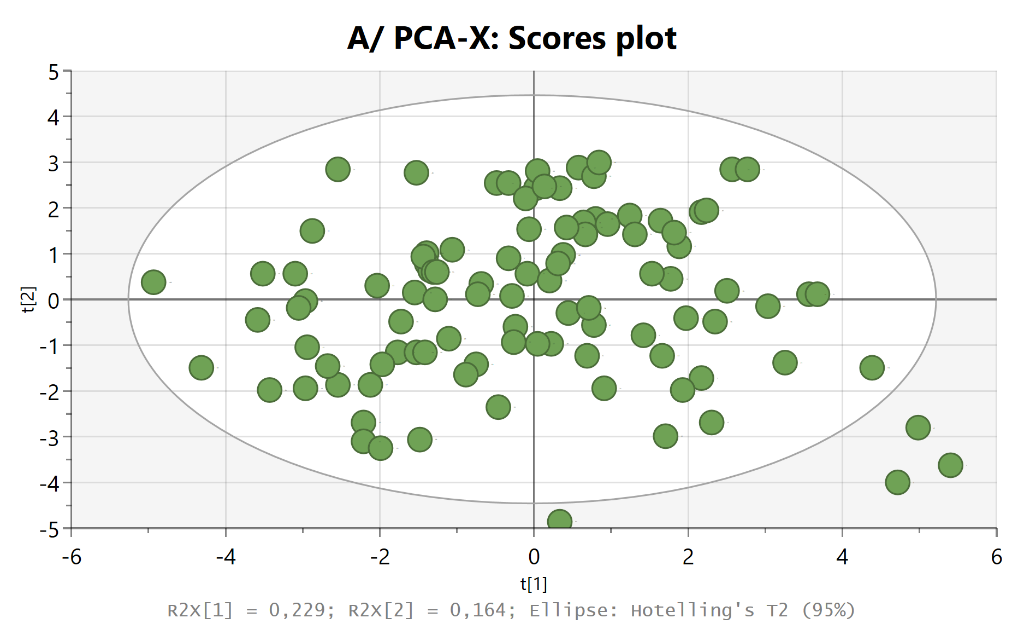


A/ Scores plot. Scores represent new “dummy” variables summarizing the x-variables. The scores are independent of each other; each score represents one patient, and the dummy variable encounters as many score vectors as there are components in the model. Ninety-eight (=96%) scores are situated within the 95% Hotelling´s T2 tolerance ellipse, with no major outliers. B/ Loadings plot. Here, the scores are weighted averages of the variables. Loadings express the dominating correlation structure of the matrix, e.g., how the independent variables relate to each other (which ones provide similar information, e.g., PINP, osteocalcin, ALKP; which ones are negatively correlated, e.g.,esRAGE to BMD z-scores; not related to each other, e.g., esRAGE and phosphates; or not well explained by the model (= those situated close to the intercept).

LS z-score, lumbar spine bone mineral density z-score; Hip z-score, left hip bone mineral density z-score; Fem. Neck z-score, left femoral neck bone mineral density z-score; TBS L2L4, lumbar spine trabecular bone (L2-L4) score; CTX, carboxy-terminal telopeptide of type I collagen; PINP, amino-terminal propeptide of human procollagen type I; CML, N^ε^-(carboxymethyl)lysine; MG-H1, methylglyoxal-derived hydroimidazolone; esRAGE, endogenous secretory receptor for advanced glycation end-products; cRAGE leaved receptor for advanced glycation end-products; ALKP, alkaline phosphatase; 25-OH vit. D, 25-hydroxy vitamin D; P, phosphates; IGF-1, insulin-like growth factor-1, E2, estradiol; AGE-Fl, advanced glycation end-products-associated fluorescence of plasma

**Supplementary Fig. S2:** The permutation plots validating the OPLS models (A/ of the hip BMD z-score; B/ of the femoral neck BMD z-score; C/ of the TBS)


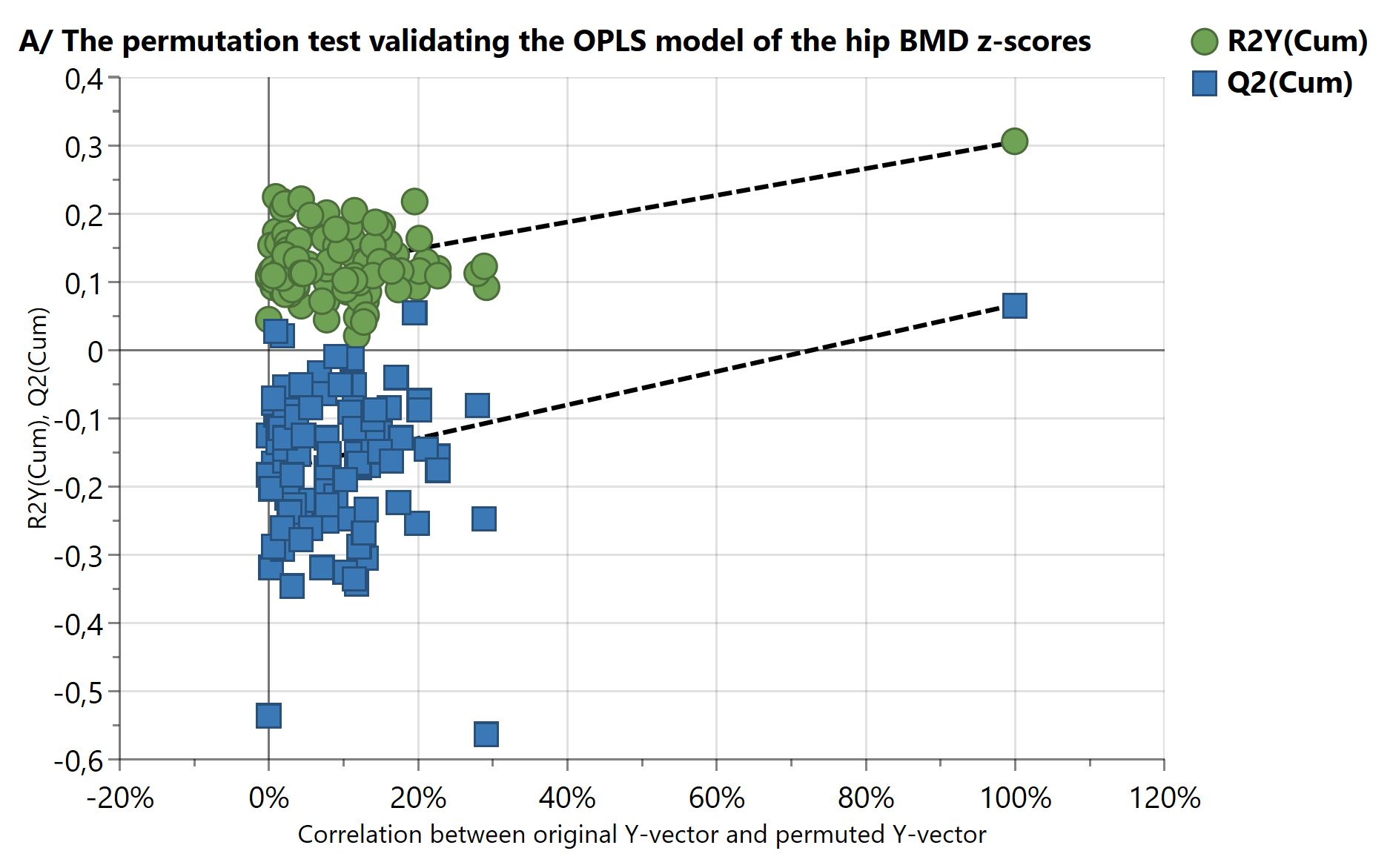


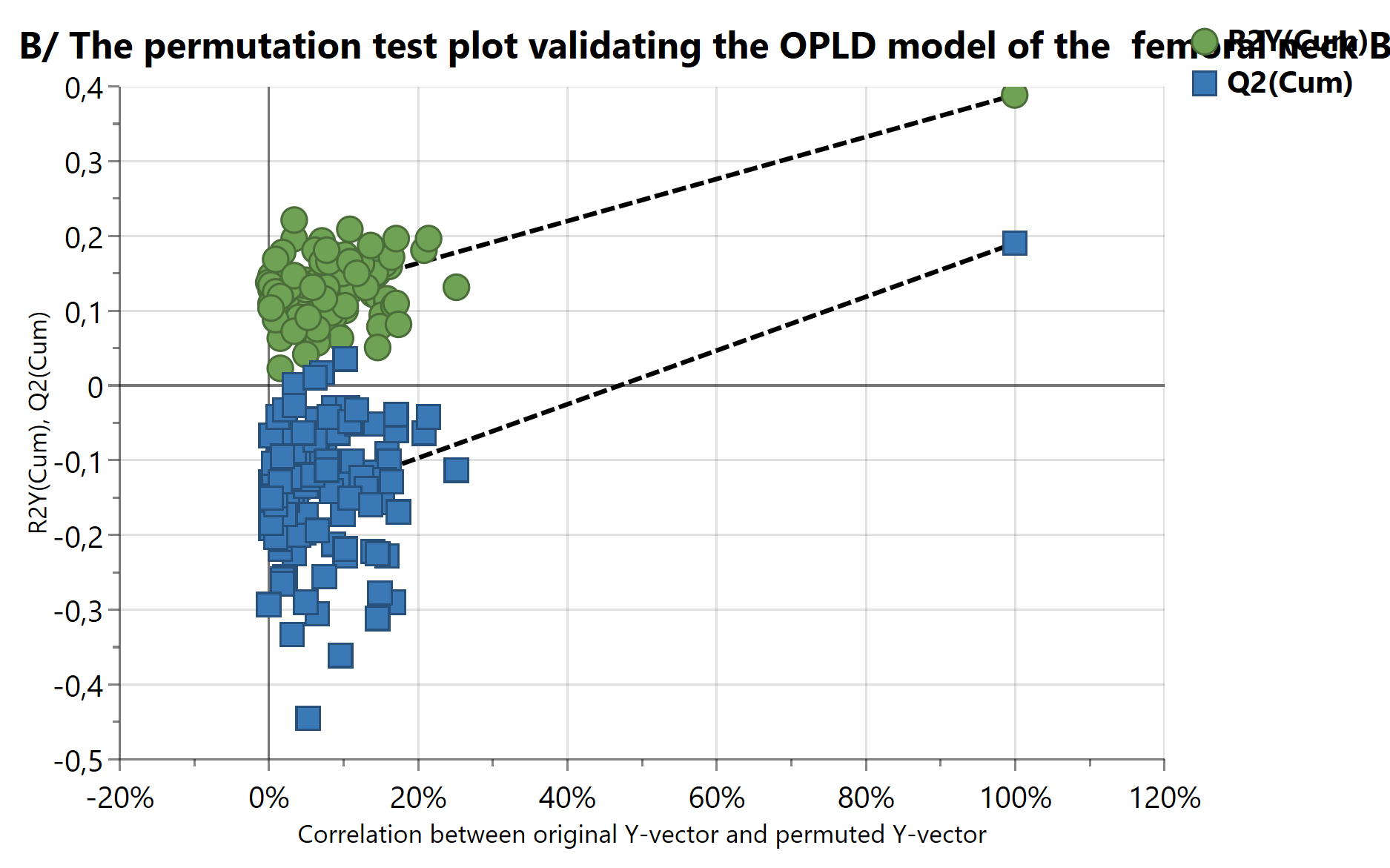


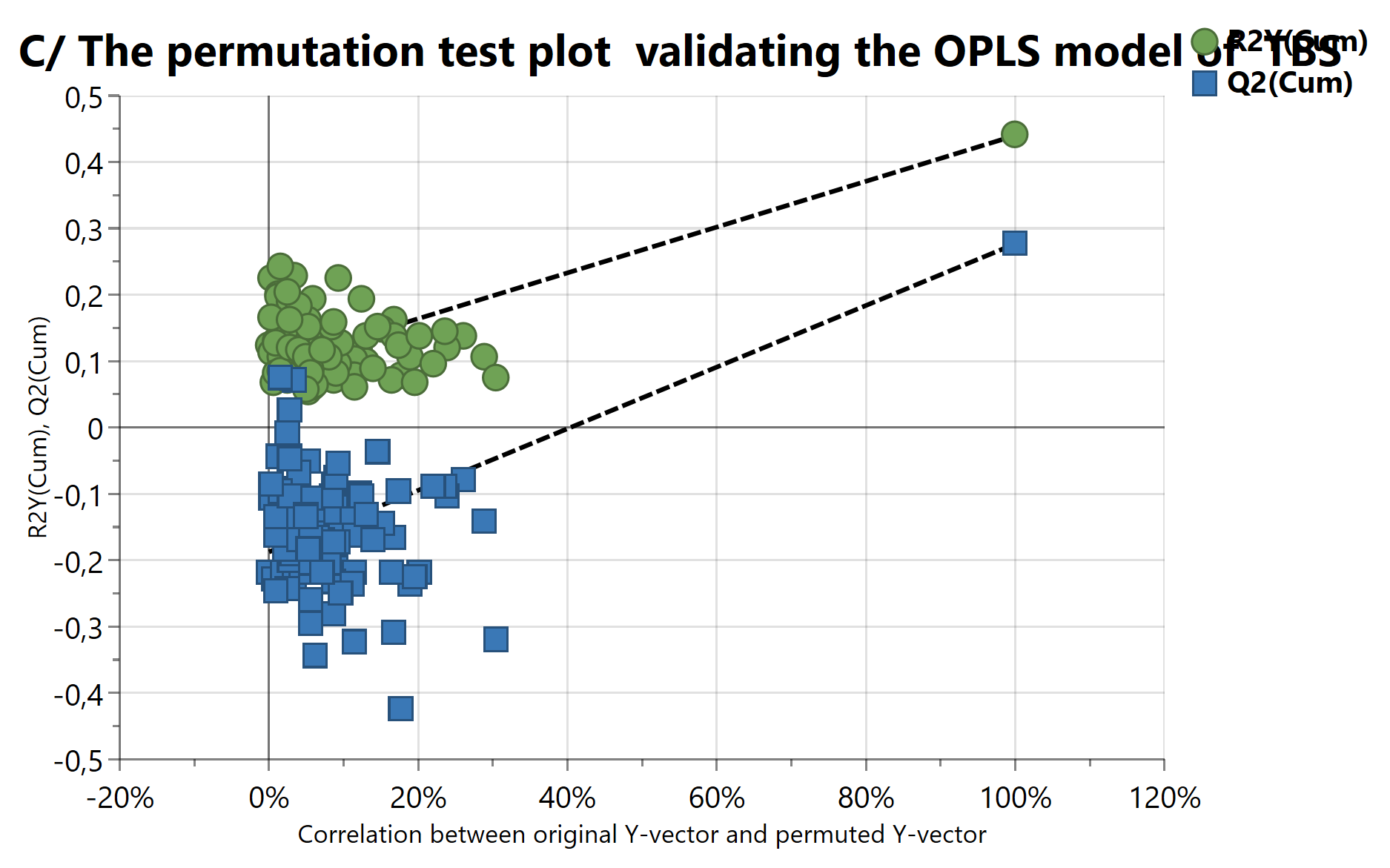


The permutation plot was evaluated to assess the model's spurious risk. Each plot shows, for a selected Y-variable, on the vertical axis far to the right, the values of R2 and Q2 for the original model and of the Y-permuted models further to the left. The horizontal axis shows the correlation between the permuted Y-vectors and the original Y-vector for the selected Y. The original Y correlates 100% with itself, defining the high point on the horizontal axis. Plots A to C strongly indicate that the original models are valid. All Q^2^ values to the left are lower than the original point to the right, and the regression line of the Q^2^ intersects the vertical axis below zero. All green R^2^ values to the left lower than the original model to the right also indicate the model's validity.

1. Čagalová A, Tichá Ľ, Gaál Kovalčíková A, Šebeková K, Podracká Ľ (2022) Bone mineral density and oxidative stress in adolescent girls with anorexia nervosa. Eur J Pediatr 181:311-321 https://doi.org/10.1007/s00431-021-04199-5

2. Munch G, Keis R, Wessels A, et al. (1997) Determination of advanced glycation end products in serum by fluorescence spectroscopy and competitive ELISA. Eur J Clin Chem Clin Biochem 35:669-677

3. Kovalčíková AG, Tichá Ľ, Šebeková K, et al. (2021) Oxidative status in plasma, urine and saliva of girls with anorexia nervosa and healthy controls: a cross-sectional study. J Eat Disord 9:54 https://doi.org/10.1186/s40337-021-00408-6
